# Beyond shape capture: The dynamic transtibial prosthetic interface and the continuing role of clinical expertise

**DOI:** 10.33137/cpoj.v9i2.47748

**Published:** 2026-06-23

**Authors:** A.G Johannesson

**Affiliations:** Össur Clinics EMEA, Stockholm, Sweden.

**Keywords:** Amputation, Additive Manufacturing, Biomechanics, CAD/CAM, Digital Workflow, Clinical Expertise, Prosthetist, Prosthetic Socket, Transtibial Prosthesis, Socket Fit, Residual Limb, Suspension Systems

## Abstract

Despite major advances in prosthetic components, digital workflows, and manufacturing technologies, substantial variability continues to exist in prosthetic interface outcomes. Socket fit, tissue tolerance, user comfort, and long-term functional success remain difficult to predict and standardize across individuals and clinical settings. While contemporary technologies such as CAD/CAM, pressure scanning, additive manufacturing, and emerging artificial intelligence applications may improve manufacturing precision and reproducibility, they do not fully address the biological and biomechanical complexities that influence interface performance. Residual limb tissues, suspension systems, gait mechanics, skin adaptation, and individual user characteristics interact dynamically throughout prosthetic use, limiting the ability of shape capture and manufacturing processes alone to determine successful outcomes. This perspective paper argues that prosthetic interface management should be viewed as a dynamic biological and biomechanical process rather than primarily a manufacturing challenge. Building upon a systems-based understanding of the interface, the paper proposes that successful outcomes emerge from the interaction between socket design, liner characteristics, suspension mechanisms, tissue biology, gait dynamics, and clinical decision-making. The paper further highlights the unique role of the prosthetist in observing long-term outcomes and emphasizes the continued importance of clinical expertise in an increasingly digital environment. Future progress in prosthetic interface management will likely depend on integrating technological innovation with rehabilitation science, biomechanics, tissue biology, and outcome-focused clinical practice.

## INTRODUCTION

Despite significant advances in prosthetic components, CAD/CAM systems, digital workflows, additive manufacturing, and emerging artificial intelligence (AI) applications, achieving consistent prosthetic interface outcomes remains a significant challenge.^1–3^ Socket fit, user comfort, tissue tolerance, and long-term satisfaction continue to vary considerably between clinicians, clinics, and users, highlighting the ongoing importance of clinical expertise and individualized care. This observation is supported by studies demonstrating substantial variability in socket design philosophies, fitting approaches, user-reported outcomes, and the prevalence of residual limb skin problems, despite advances in prosthetic technology and manufacturing methods.^4^

The growing adoption of digital technologies has created expectations that many of these challenges may eventually be solved through improved scanning, modeling, and manufacturing processes.^3^ However, the persistence of variability suggests that prosthetic interface management involves factors that extend beyond the accurate capture and reproduction of shape.^5^

This challenge is further compounded by the absence of universally adopted and standardized prosthetic fit outcome measures. While considerable effort has been devoted to improving socket design, manufacturing precision, and interface technologies, there is still no universally accepted method for objectively defining a successful fit. The ISO 13405 series provides an important framework for the classification and description of prosthetic components, including socket design, supporting greater consistency in terminology, reporting, and evaluation within prosthetics.^6^ However, despite these advances, the assessment of socket fit remains influenced by clinical judgment, user perception, functional performance, and individual adaptation. Consequently, the question of what constitutes a “good” socket fit continues to be open to interpretation, ensuring that discussions regarding the ideal interface are likely to persist regardless of technological advances.

This perspective paper argues that the prosthetic interface should be viewed as a dynamic biological and biomechanical system rather than a static manufacturing problem. The interaction between tissue mechanics, suspension, gait dynamics, skin adaptation, and clinical decision-making may explain why interface outcomes continue to vary despite technological advances.

### The Historical Evolution of Interface Management

Prosthetics and orthotics evolved from craft-based professions in which success depended heavily on practical experience, repeated adjustments, and direct interaction with the user. Early practitioners developed solutions through observation, iterative problem-solving, and continuous refinement of fit and function.^7^

The introduction of modular component systems, industrial manufacturing processes, and digital design technologies has substantially transformed prosthetic practice. As fabrication methods became increasingly standardized, the role of the prosthetist evolved from primarily manufacturingfocused activities toward clinical assessment, interface management, alignment optimization, rehabilitation support, and long-term outcome management.^6,7^

While these developments have improved efficiency and reproducibility, successful prosthetic rehabilitation continues to depend heavily on clinical reasoning, individualized decision-making, and adaptation to the biological realities of each user.

### The Prosthetic Interface Is Not a Static Shape

In this paper, the Interface Triad is defined as the interdependent relationship between socket geometry, liner behavior, and suspension strategy, all operating within the biological context of the residual limb; this concept is developed further in the article.

Recent discussions have suggested that CAD software could move beyond traditional uniform socket reductions and incorporate tissue-specific modification strategies based on regional tissue characteristics and loading tolerance. Such an approach would be more consistent with the principles underlying hydrostatic casting techniques,^8^ which aim to achieve differential tissue compression during socket formation.

This concept is attractive because soft tissues and bony structures respond differently to loading. Areas with thick, compliant tissue compress more readily than areas with limited soft tissue coverage. Pressure scanning may therefore appear to offer a more physiologically accurate representation of the residual limb than conventional digital reduction methods.^9^

However, a fundamental question remains: Which shape should be captured? The shape obtained during pressure casting or pressure scanning represents only a single moment in time, typically during stance phase when the residual limb is loaded and the patient is in an upright position. A prosthetic socket must function throughout the entire gait cycle.

During heel strike, substantial forces are transferred through the residual limb. The distal tibia moves forward and downward within the surrounding soft tissues as load is applied. As the body progresses toward toe-off, the loading pattern changes and the residual limb rotates within the socket. During swing phase, loading decreases substantially, while muscle activity and tissue positions change once again.^10^

At the same time, the anatomy around the knee is constantly changing. During knee extension, the femoral condyles move anteriorly, and the patellar region changes shape. During knee flexion, the medial hamstring tendons become increasingly prominent and require additional space.^11^ These anatomical changes have traditionally required modifications to posterior trimlines and have contributed to the development of alternative trimline concepts such as Söderberg's trimline.^12^

The challenge, therefore, is not simply to determine the correct shape. Rather, it is to determine which shape is relevant at which point in the gait cycle and how a single prosthetic interface can accommodate these continuously changing conditions.

### Suspension, Tissue Mechanics, and Skin Adaptation

Suspension introduces an additional layer of complexity. Distal suspension systems, including pin-lock and lanyard designs, provide secure suspension but may also generate repetitive distal distraction forces and increased pistoning during gait. Over time, these mechanical forces have been associated with residual limb volume fluctuations, distal tissue stretching, discomfort, skin problems, and reduced prosthetic tolerance compared with suspension systems that provide greater control of limb movement and volume management.^13^

Vacuum-assisted suspension systems attempt to address some of these challenges by reducing pistoning, improving volume management, and creating a more intimate connection between the residual limb and the socket.^14^ Recent suspension concepts have combined mechanical locking mechanisms with Seal-In and vacuum-assisted suspension technologies. The design aims to reduce pistoning and improve rotational control while maintaining the perceived security of a mechanical lock and supporting residual limb volume management.

The importance of suspension highlights a broader principle: socket shape alone does not determine prosthetic interface performance. Interface performance is influenced by the interaction between socket design, suspension method, tissue behavior, gait mechanics, and long-term biological adaptation.

Recent research further challenges traditional assumptions regarding tissue adaptation. Hayes et al. demonstrated that residual limb skin may become softer, exhibit increased friction, demonstrate reduced barrier function, and show evidence of chronic low-grade inflammation. Residual limb tissues were also found to be more compliant than both normal skin and heel skin on the contralateral limb.^15^ These findings suggest that tissues may not necessarily become stronger through long-term prosthetic loading. In some cases, they may become more vulnerable to injury. Importantly, increased friction creates greater shear forces within the interface. While prosthetic socket design has historically focused on pressure management, these findings suggest that friction and shear may be equally important contributors to skin breakdown and discomfort.^15^

### The Interface Triad: Socket, Liner, and Suspension

A recurring theme throughout interface management is that individual components cannot be evaluated independently. The socket, liner, and suspension components function as an interconnected interface system. Changes to one component inevitably influence the behavior of the others. Consequently, interface performance cannot be attributed to any single component in isolation but rather to the interaction between socket design, liner characteristics, suspension mechanisms, tissue biology, and user-specific gait dynamics.

This systems-based perspective is consistent with the terminology and conceptual framework reflected in international standards such as the upcoming revised ISO 13504 series, which recognize the multidimensional nature of prosthetic outcomes. Building upon this foundation, the Interface Triad is proposed as a conceptual framework for understanding interface performance and long-term prosthetic outcomes.

Rather than evaluating socket shape, liner selection, or suspension method separately, clinicians and researchers should consider how these elements interact with tissue biology and gait mechanics. The Interface Triad emphasizes that successful interface management emerges from the dynamic interaction between the socket, liner, and suspension system, rather than from the optimization of any individual component alone.

### Why Clinical Expertise Still Matters

Digital technologies may improve reproducibility, documentation, and manufacturing consistency. However, they do not eliminate the need for clinical interpretation.

One of the unique perspectives that prosthetists bring to amputation care is their long-term involvement in the rehabilitation process. While surgeons are responsible for the critical decisions made before and during amputation, they often have limited opportunities to observe the long-term consequences of these decisions on prosthetic fitting, mobility, comfort, and tissue tolerance. In contrast, prosthetists may follow individuals for decades, gaining direct insight into how surgical techniques, residual limb characteristics, and postoperative management influence prosthetic outcomes over time. This perspective highlights the importance of involving prosthetists early in the amputation pathway, allowing surgical and rehabilitation decisions to be informed not only by immediate clinical needs but also by their potential impact on lifelong prosthetic function and quality of life.^16^

The prosthetist remains responsible for understanding tissue behavior, gait mechanics, skin health, user goals, suspension effects, and long-term adaptation. These variables often cannot be fully captured through scanning technologies or reduction algorithms alone.

Consequently, successful interface management remains dependent upon clinical expertise. As interface systems become increasingly sophisticated, the involvement of experienced certified prosthetists in research, development, and clinical implementation may become even more important, not less.

### Future Directions

Future research should focus on understanding the dynamic interaction between tissue biology, suspension, gait mechanics, and interface design. Greater emphasis should also be placed on outcome evaluation, skin health, friction management, and long-term tissue adaptation. Most importantly, future technological developments should aim to support clinical decision-making rather than replace it.

Despite decades of advances in socket fabrication, liner technology, suspension systems, and digital workflows, no universally accepted definition of an optimal prosthetic interface currently exists. This observation alone suggests that successful interface management involves factors that extend beyond manufacturing precision and shape reproduction.

## CONCLUSION

The prosthetic interface should not be viewed primarily as a shape-capture or manufacturing challenge. Rather, it is a dynamic biological and biomechanical system influenced by tissue mechanics, suspension, gait dynamics, skin adaptation, rehabilitation processes, and clinical decision-making. Variability in prosthetic outcomes is therefore unlikely to be explained by any single technological, clinical, or organizational factor.

While digital technologies, CAD/CAM systems, additive manufacturing, and emerging applications of artificial intelligence offer important opportunities to improve consistency, documentation, and manufacturing efficiency, they do not eliminate the complexity of the residual limb or its interaction with the prosthetic socket. The challenge is not simply to identify the “correct” shape, but to understand how a dynamic interface behaves throughout the gait cycle and over the lifetime of the prosthesis user.

This perspective has argued that successful interface management depends on understanding the interaction between the socket, liner, and suspension system, together with the biological response of the residual limb. Future progress will therefore require stronger integration of clinical expertise, tissue biology, biomechanics, rehabilitation science, and technological innovation.

The future of transtibial interface care will likely depend not on replacing clinical expertise with technology, but on using technology to support more consistent, transparent, and evidence-informed clinical decision-making. Equally important, prosthetic rehabilitation remains a profoundly human process. Individuals adapting to limb loss often face significant physical, psychological, and social challenges. As a result, the relationship between the prosthesis user and the prosthetist will continue to play a central role in achieving successful long-term outcomes. Technology may enhance care, but it cannot replace the clinical judgement, empathy, and individualized support that remain fundamental to effective rehabilitation.

## Figures and Tables

**Figure:**
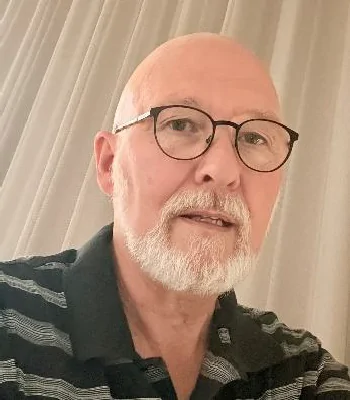


## AUTHORS BIOGRAPHY

**Dr. Anton G. Johannesson** is a European-certified Prosthetist and Orthotist, graduating from the University of Jönköping, Sweden, in 1990. He earned his PhD in Orthopaedics from Lund University in 2009, with a thesis on lower limb amputation in patients with vascular disease. His research has contributed to improved understanding of surgical technique, postoperative care, early rehabilitation, and prosthetic outcomes.

Dr. Johannesson founded and served as CEO of Orthopedic Technology Inc. in Kristianstad, Sweden. He later served as Clinical Manager of Prosthetics at TeamOlmed Clinics in Sweden from 2014 to 2023, where he focused on clinical innovation, education, research, and collaboration with Össur R&D. Since June 2023, he has worked as Director of Clinical Development, Prosthetics, with a continued focus on advancing clinical practice, prosthetic technology, and education.

His professional and research work has centered on lower limb prosthetics, transtibial amputation care, prosthetic socket design, postoperative compression, early prosthetic fitting, and outcomebased rehabilitation. He has authored and co-authored more than 20 peer-reviewed scientific publications and has been actively involved in international education and clinical training, including support for prosthetics and orthotics development in Ukraine.

Dr. Johannesson is also active in international standardization and professional organizations. He serves as Convenor of ISO/TC 168 Working Groups 1 and 2 in Prosthetics and Orthotics and has held leadership roles within the International Society for Prosthetics and Orthotics. His work combines clinical experience, research, education, and standardization with the aim of improving prosthetic care and long-term outcomes for people with limb loss.
